# High-transmission spectrometer for rapid resonant inelastic soft X-ray scattering (rRIXS) maps

**DOI:** 10.1107/S160057752400804X

**Published:** 2024-09-30

**Authors:** Lothar Weinhardt, Constantin Wansorra, Ralph Steininger, Thomas Spangenberg, Dirk Hauschild, Clemens Heske

**Affiliations:** a Institute for Photon Science and Synchrotron Radiation (IPS) Karlsruhe Institute of Technology (KIT) Kaiserstr. 12 76131Karlsruhe Germany; b Institute for Chemical Technology and Polymer Chemistry (ITCP) Karlsruhe Institute of Technology (KIT) Kaiserstr. 12 76131Karlsruhe Germany; c Department of Chemistry and Biochemistry University of Nevada, Las Vegas (UNLV) 4505 Maryland Parkway Las Vegas NV89154-4003 USA; Bhabha Atomic Research Centre, India

**Keywords:** soft X-ray spectrometer, high-transmission, rapid RIXS, XES, rRIXS map

## Abstract

The design and first results of a high-transmission soft X-ray spectrometer operated at the X-SPEC double-undulator beamline of the KIT Light Source are presented. As a unique feature, particular emphasis was placed on optimizing the spectrometer transmission by maximizing the solid angle and the efficiencies of spectrometer gratings and detector. A CMOS detector, optimized for soft X-rays, allows for quantum efficiencies of 90% or above over the full energy range of the spectrometer, while simultaneously offering short readout times. Combining an optimized control system at the X-SPEC beamline with continuous energy scans (as opposed to step scans), the high transmission of the spectrometer, and the fast readout of the CMOS camera, enable the collection of entire rapid resonant inelastic soft X-ray scattering maps in less than 1 min. Series of spectra at a fixed energy can be taken with a frequency of up to 5 Hz. Furthermore, the use of higher-order reflections allows a very wide energy range (45 to 2000 eV) to be covered with only two blazed gratings, while keeping the efficiency high and the resolving power *E*/Δ*E* above 1500 and 3000 with low- and high-energy gratings, respectively.

## Abstract 2

A high-transmission soft X-ray spectrometer has been developed and commissioned at the X-SPEC beamline of the KIT Light Source. With this spectrometer, single spectra can be measured with exposure times well below 1 s, allowing the rapid collection of full resonant inelastic soft X-ray scattering maps in less than one minute.

## 1. Introduction

Soft X-ray emission spectroscopy (XES) and, when performed with resonant excitation, resonant inelastic soft X-ray scattering (RIXS) are very powerful techniques to probe many different aspects of the electronic structure of materials. Both XES and RIXS are ‘local’ techniques due to the selection of a specific core orbital, allowing for a variety of studies, including on chemical bonding (O’Bryan & Skinner, 1940 ▸; Meisel *et al.*, 1968 ▸; Meyer *et al.*, 2014 ▸; Seitz *et al.*, 2022 ▸; Weinhardt, Hau­schild *et al.*, 2021 ▸; Li *et al.*, 2023 ▸), band structure (Johnson & Ma, 1994 ▸; Carlisle *et al.*, 1995 ▸; Eich *et al.*, 2006 ▸), vibrational structure in molecular systems (Werme *et al.*, 1973 ▸; Hennies *et al.*, 2010 ▸; Rubensson *et al.*, 2015 ▸; Ertan *et al.*, 2018 ▸), as well as elementary excitations (charge transfer, *d*–*d*, magnons, phonons) (Chiuzbăian *et al.*, 2005 ▸; Ghiringhelli, Matsubara *et al.*, 2006 ▸; Ament *et al.*, 2011 ▸; Schlappa *et al.*, 2012 ▸; Vale *et al.*, 2019 ▸; Lu *et al.*, 2021 ▸).

Fueled by this versatility, XES and RIXS have gained an important role in synchrotron spectroscopy, with many new instruments coming online in the past two decades. Generally, the experimental setups have been developed in two opposing directions: high resolution and high transmission. The former is particularly important for studying elementary excitations that can require a resolution of a few tens of meV. This has been achieved by building very long (>5 m) spectrometers (Ghiringhelli, Piazzalunga *et al.*, 2006 ▸; Chiuzbăian *et al.*, 2014 ▸; Dvorak *et al.*, 2016 ▸; Zhou *et al.*, 2022 ▸), but the price to pay is a comparably low transmission that leads to long exposure/acquisition times.

While this is affordable for many questions in fundamental research, this can be a severe limitation for the investigation of applied material systems. Many samples, in particular organic materials, can already be damaged by soft X-rays after short exposure (Zubavichus *et al.*, 2004 ▸; Weinhardt *et al.*, 2008 ▸, 2019 ▸; Wilks *et al.*, 2009 ▸; Léon *et al.*, 2017 ▸; Roychoudhury *et al.*, 2021 ▸), which requires the minimization of the radiation dose. Furthermore, the element of interest might occur in a very low concentration, which requires an efficient detection. And finally, the past decade has seen a growing interest in *operando* studies using soft X-rays, and suitable sample environments have been developed (Heske *et al.*, 2003 ▸; Blum *et al.*, 2009 ▸; Nagasaka *et al.*, 2010 ▸; Schwanke *et al.*, 2014 ▸). In these experiments, quick measurements are required to monitor changes of the sample as a function of time. This has motivated the development of high-transmission spectrometers, for which several strategies have been used. While earlier designs (Nordgren & Nyholm, 1986 ▸; Callcott *et al.*, 1986 ▸; Shin *et al.*, 1995 ▸; Dallera *et al.*, 1996 ▸) used an entrance slit to define the source spot, current spectrometers significantly increase the solid angle by using slit-less designs with the source spot defined by the beam spot on the sample (Cocco *et al.*, 2004 ▸; Chuang *et al.*, 2005 ▸; Tokushima *et al.*, 2006 ▸; Ghiringhelli, Piazzalunga *et al.*, 2006 ▸; Fuchs *et al.*, 2009 ▸; Chiuzbăian *et al.*, 2014 ▸; Dvorak *et al.*, 2016 ▸; Qiao *et al.*, 2017 ▸; Zhou *et al.*, 2022 ▸). This must be used with some caution in case of radiation-sensitive samples, since smaller sample spots also increase the radiation dose.

The development of a short, slit-less spectrometer with spherical mirror, plane variable-line-space (VLS) grating, and a CCD detector in normal incidence by some of the authors achieved a significant improvement in spectrometer transmission collecting spectra with acquisition times as short as 10 s (Fuchs *et al.*, 2009 ▸). This allowed the development of the soft X-ray RIXS map (Fuchs *et al.*, 2009 ▸; Weinhardt *et al.*, 2009 ▸), where a RIXS spectrum is measured for every point in a regular X-ray absorption scan; the emission intensity is then plotted color-coded as a function of excitation and emission energies. Typical measurement times for such a RIXS map are of the order of a few tens of minutes. Other spectrometer designs use transmission gratings (Hatsui *et al.*, 2005 ▸; Yamane *et al.*, 2013 ▸) or transmission off-axis zone plates (Marschall *et al.*, 2017 ▸; Schunck *et al.*, 2021 ▸) to further increase the solid collection angle. By opening the beamline exit slit and moving the beamline monochromator focus from the exit slit to the sample, some additional degree of parallelization can be achieved, allowing an excitation energy range of a few eV to be collected simultaneously with suitable imaging optics of the spectrometer (Warwick *et al.*, 2014 ▸; Chuang *et al.*, 2020 ▸; Marschall *et al.*, 2017 ▸; Schunck *et al.*, 2021 ▸). Combining this parallel collection scheme with transmission off-axis zone plates, a section of a RIXS map can be collected ‘in one shot’ within approximately 10 min (Marschall *et al.*, 2017 ▸). The energy window of this RIXS map section is limited in excitation energy by the bandwidth of the beamline (of a few eV) and the energy window in focus for one setting of the spectrometer (also a few eV) (Marschall *et al.*, 2017 ▸). A very different strategy for high transmission is the use of an energy-dispersive design instead of the wavelength-dispersive approaches discussed above. This promises very high efficiency with large solid angle and is insensitive to the spot size on the sample. In recent years, this approach has been steadily improved and a first instrument is in operation (Lee *et al.*, 2019 ▸), but its energy resolution of 1.5 eV is still inferior to their wavelength-dispersive counterparts and maximal count rates are limited due to long dead-times. A second, energy-dispersive strategy is the ‘photoelectron spectrometry for analysis of X-rays’ (PAX) that uses a material converting X-rays into photoelectrons, which are then detected with a conventional electron analyzer; however, only a first demonstration experiment has been reported to date (Dakovski *et al.*, 2017 ▸).

In this paper, we present a very high transmission soft X-ray spectrometer at the X-SPEC double-undulator beamline (Weinhardt, Steininger *et al.*, 2021 ▸) at the KIT Light Source that allows single spectra to be measured in a fraction of a second, full RIXS maps in half a minute (henceforth termed rRIXS, for ‘rapid RIXS’), and achieves a resolving power of 1500 or better over its wide energy range from 45 to 2000 eV.

## 2. Design considerations and optical layout

The presented soft X-ray spectrometer is aimed for use in the investigation of applied material systems under ultra-high vacuum (UHV), *in situ* and *operando* conditions. As outlined above, this requires maximizing the spectrometer transmission while still maintaining the required experimental resolution. For chemical bonding properties and electronic band structure information, an energy resolution of 0.2–0.5 eV is sufficient in most cases, which can also resolve *d*–*d* and vibrational excitations in some cases. As the final requirement in our case, the X-ray spectrometer needs to cover energies from 45 to 2000 eV to include the entire soft X-ray energy range of the X-SPEC beamline (Weinhardt, Steininger *et al.*, 2021 ▸) and allow for Li *K* XES (*i.e.* with non-resonant excitation). The X-SPEC beamline has two experimental stations for studies under UHV and *in situ*/*operando* conditions at atmospheric pressures or above, respectively. Each of these endstations is equipped with one X-ray spectrometer of the design discussed in this paper.

To fulfill all of the discussed requirements, our spectrometer uses a slit-less design with spherical VLS gratings (one for low and one for high energies) as the single optical element, coupled with a soft X-ray CMOS camera. The gratings for low and high energies have central groove densities of 600 and 1200 lines mm^−1^, with energy ranges of 45–800 and 450–2000 eV, respectively. To cover these large energy ranges efficiently, we make extensive use of higher diffraction orders (Fuchs *et al.*, 2009 ▸), as discussed below. In general, three main factors can be exploited to optimize the X-ray spectrometer transmission: the use of parallelization, the acceptance angle, and the efficiency of the optics and the detector.

**Figure 1:**
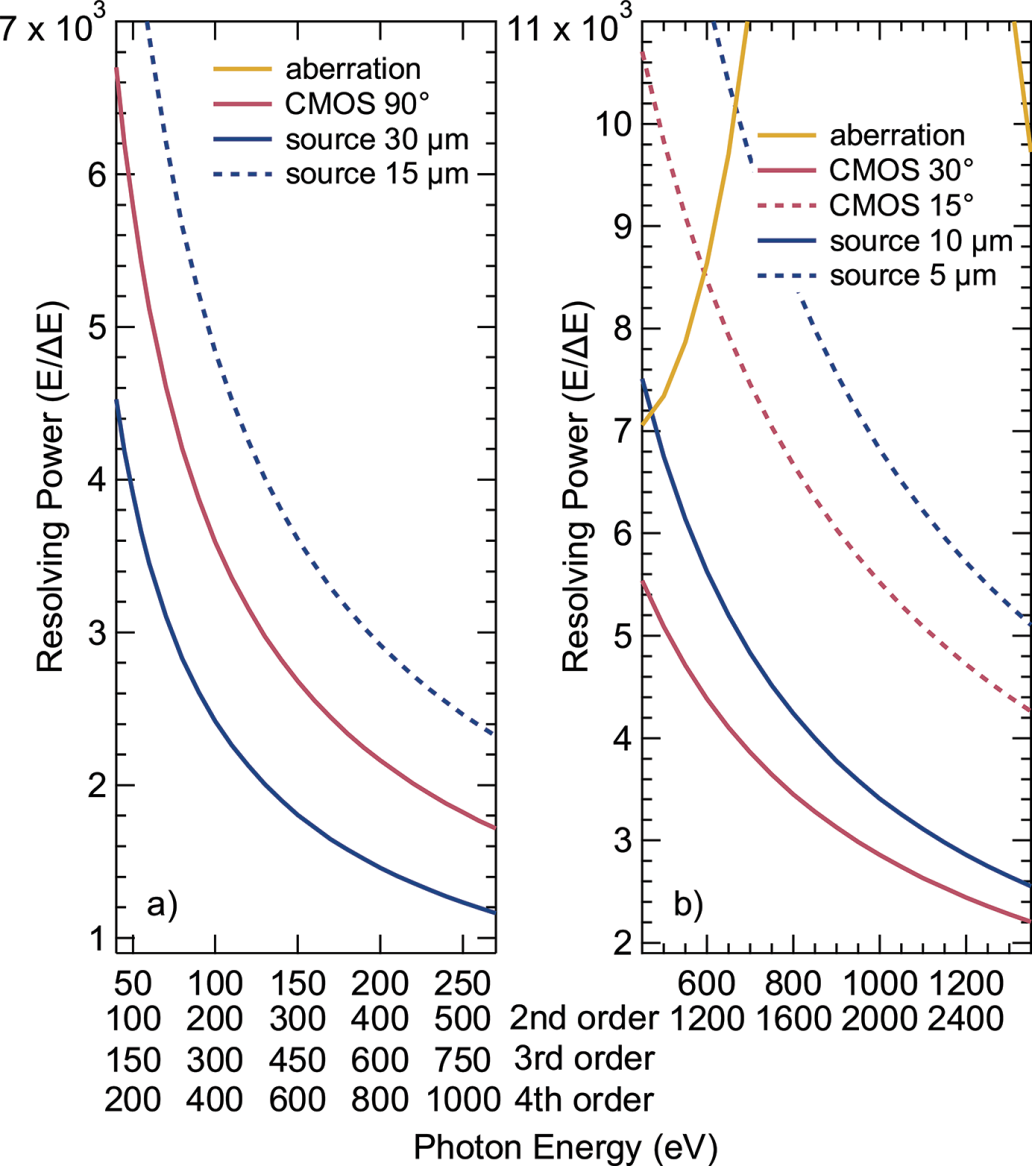
The different contributions to the spectrometer resolving power for (*a*) LEG and (*b*) HEG as a function of photon energy (given in different orders on the abscissa). Variations of source spot size (blue) and CMOS incidence angle (red) are also shown. Note that aberrations (yellow) do not play any role for the LEG and thus do not appear in the shown resolving power range in (*a*).

To measure all photon energies of a spectrum in parallel with an area detector, care must be taken that aberrations are sufficiently corrected in the relevant energy window. To achieve this, the VLS parameters and radii of the gratings were optimized numerically using the procedure by Strocov *et al.*, as implemented in the *GeneVLS* package (Strocov *et al.*, 2011 ▸), and checked by ray-tracing using the *RAY* program (Schäfers, 2008 ▸). All optical parameters of the spectrometer are listed in Table 1 ▸. For the low-energy grating (LEG), parameters were optimized for an energy of 180 eV, and for the high-energy grating (HEG) for an energy of 1000 eV. For other center energies on the detector, aberrations can be minimized by changing the grating angle and grating–detector distance to values optimized using *TraceVLS* (Strocov *et al.*, 2011 ▸). With this, aberrations do not play any role over the full energy range of the LEG, and only influence the overall resolving power for the HEG at lower energies. This can be seen in Fig. 1 ▸, where the contributions of aberrations, source spot size and CMOS pixel size are illustrated. Depending on the beamline settings, the resolving power is limited by the source spot on the sample (with a minimum of ∼5 µm for narrow beamline exit slits) or the effective pixel size (approximated with 20 µm) of the CMOS detector. It decreases with increasing photon energy. For the LEG, source sizes of 30 µm are sufficient to keep the resolving power *E*/Δ*E* above 1500. This allows large exit slits of the beamline to be used to maximize the photon flux. The smaller source sizes and thus beamline exit slits required for the HEG match the need to keep the resolving power of the beamline in line with that of the spectrometer of *E*/Δ*E* above 3000 for RIXS experiments. Limited by the mechanical movement of the detector, the LEG covers an energy range from 45 eV to 270 eV in first diffraction order, while the center energy of the HEG in first diffraction order can be tuned from 450 to 1350 eV. The remaining energies are covered in higher orders (up to fourth order for the LEG and second order for the HEG), as illustrated by the respective energy scales on the abscissa in Fig. 1 ▸. Besides limiting the necessary detector movement, using higher orders allows to avoid the decrease in resolving power at higher energies due to source and detector broadening. For the LEG, the detector can be operated in normal incidence, while it needs to be tilted to more grazing incidence for the HEG to reduce the effective pixel size and be parallel to the focal plane.

As described in the *Introduction*[Sec sec1], spectrometer designs exist that also measure a (small) range of incoming photon energies in parallel. However, this needs additional optical elements and only works with very homogeneous samples. The latter is often not the case for applied material systems, and thus this option is not used for our spectrometer.

To optimize the acceptance angle in the non-dispersive direction, the total length of the spectrometer was minimized (1.4–1.8 m depending on the center energy). For maximal acceptance in the dispersive direction, the gratings were placed as close as possible to the sample spot (400 mm) with their length maximized (150 mm), while still meeting the requirements on energy resolution. Some spectrometer designs furthermore use collection mirrors to increase the acceptance angle in the non-dispersive direction (Tokushima *et al.*, 2011 ▸; Dvorak *et al.*, 2016 ▸; Zhou *et al.*, 2022 ▸). The gain of this approach is highest for very long spectrometers, while short spectrometers will not profit as much. For this reason, the additional cost, and the more complex alignment, no collection mirrors were used in our design. Overall, our spectrometer has, depending on energy, acceptance angles (non-dispersive × dispersive) between 12.5 mrad × 22.7 mrad and 16.1 mrad × 12.2 mrad for the LEG, and 12.5 mrad × 6.9 mrad and 16.1 mrad × 6.6 mrad for the HEG.

**Figure 2:**
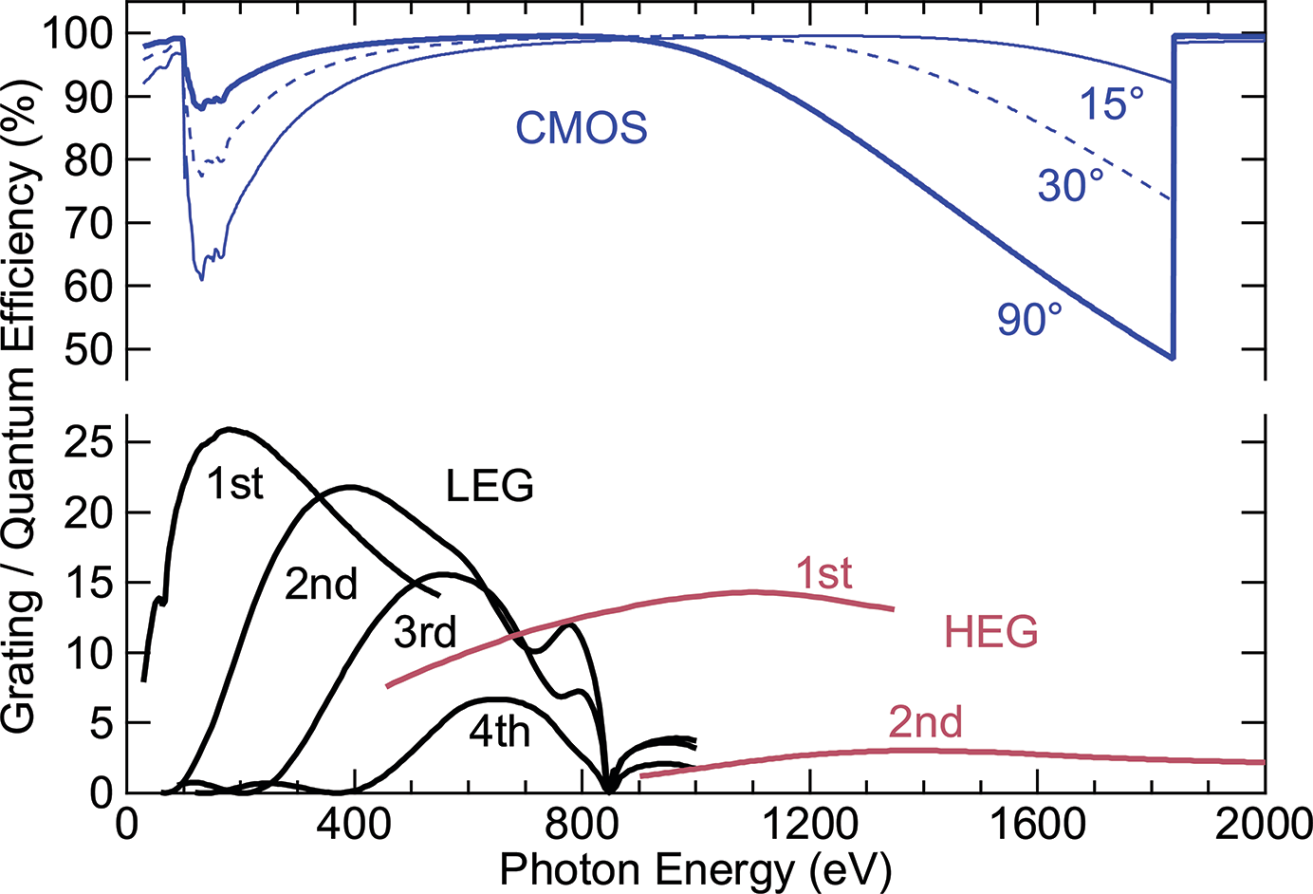
Simulated grating and CMOS sensor quantum efficiencies as a function of photon energy. Grating efficiencies are given for the LEG (black) and the HEG (red) in different orders, as indicated in the graph. The CMOS quantum efficiency (blue) is given for three different incidence angles, with 90° corresponding to normal incidence.

With only one optical element, reflection losses are reduced compared with designs with several optical elements. For the LEG coating, we chose nickel, which gives the highest and most uniform reflectivity for energies up to 800 eV, with incident angles between 2.9° (around 50 eV) and 1.8° (around 270 eV) in first order. For the HEG, the incidence angle is close to 1° for all energies, and a gold coating was chosen. The reflectivity, in particular for the higher orders, is further maximized by using blazed gratings. The blaze angles of the two gratings were optimized to 1.7° (LEG) and 0.9° (HEG) by simulating the grating efficiency with the *REFLEC* software (Schäfers, 2008 ▸) to give the best performance for the respective energy ranges. The simulated grating efficiencies are shown in Fig. 2 ▸ as a function of photon energy, grating and diffraction order. Using higher orders allows to stay close to the optimal blaze angle over the full energy range. For the LEG, this leads to very high efficiencies in all orders, while going to second order costs a factor of about four for the HEG (necessary when a high resolving power is required, see above). Gratings were manufactured by Diffraction Optics Solutions (DIOS) with the parameters listed in Table 1 ▸, using mechanical ruling and ion milling to adjust the blaze angle.

As detector, we used a back-illuminated CMOS sensor with 2048 × 204 pixels of 11 µm × 11 µm from Gpixel Inc. optimized for EUV and soft X-rays (Harada, Teranishi, Watanabe, Zhou, Bogaerts *et al.*, 2019 ▸). For energies below 1000 eV, this sensor has a quantum efficiency of 90% or above in normal incidence, as has been experimentally characterized and simulated (Harada, Teranishi, Watanabe, Zhou, Bogaerts & Wang, 2019 ▸). Using the same simulation approach with the thickness values of the ‘dead-layer’ (5 nm) and the active layer (9.5 µm) (from Harada, Teranishi, Watanabe, Zhou, Bogaerts & Wang, 2019 ▸), we have simulated the quantum efficiency for photon energies up to 2000 eV and different incidence angles in Fig. 2 ▸. In this approach, the quantum efficiencies are estimated by the product of the transmission of the ‘dead layer’ and the absorption of the active layer (Harada, Teranishi, Watanabe, Zhou, Yang *et al.*, 2019 ▸). X-ray attenuation lengths were retrieved from the CXRO X-ray database (Henke *et al.*, 1993 ▸). For normal incidence, the quantum efficiency drops from close to 100% at 1000 eV to ∼50% just below the Si *K*-edge at ∼1.8 keV. This is mitigated by using the sensor in more grazing incidence with the HEG (which is also necessary to achieve the desired resolving power, as discussed above) with simulated quantum efficiencies above 90% for energies above 600 eV and 15° incidence. Currently, an AXIS-SXRF-EUV camera with the EUV optimized CMOS sensor is installed. In this camera, the sensor is fixed in the flange plane, and we can vary the incidence angle only between ∼75 and 90° incidence. For optimal operation with the HEG, we will upgrade to a custom-built camera with the same sensor that can be rotated over the full range between 0° and 90°. Besides the very high quantum efficiency, the CMOS sensor allows for much higher frame rates (up to 48 Hz) as compared with a CCD camera. As will be shown below, this is essential for using the full potential of the presented spectrometers.

## 3. Control system

To profit from the very short acquisition times possible with our spectrometer, a very efficient and fast data collection is necessary. In particular, the dead-time per data point must be kept as short as possible, which is challenging for the control system and, during energy scans, the beamline mechanics. The spectrometer is integrated with a Tango server (Götz *et al.*, 2003 ▸) into the X-SPEC control system, which is based on *SPEC* (Certified Scientific Software, Cambridge, USA). *SPEC* allows measurements with acquisition dead-times of currently 150–170 ms at X-SPEC, and the use of macros and easy interfacing to other software makes it possible to automatize complex measurement programs. However, during standard stepwise energy scans, dead-times are dominated by the undulator and monochromator movements, which can take a few seconds per energy step. At X-SPEC, we have thus implemented energy scans in which undulator and monochromator are run continuously and in parallel with an adjustable energy change per time. With this, we can achieve speeds of up to 2 eV per second (depending on the energy range), only limited by the need of precise motions with the long lever arms of the monochromator mechanics.

## 4. Rapid RIXS maps of boron nitride

Since their introduction (Fuchs *et al.*, 2008 ▸, 2009 ▸; Weinhardt *et al.*, 2009 ▸), soft X-ray RIXS maps have become a powerful tool, giving – arguably – the most complete electronic structure depiction possible with a single measurement. RIXS maps have been used for fundamental studies of solids (Weinhardt *et al.*, 2009 ▸; Schlappa *et al.*, 2012 ▸; Huang *et al.*, 2017 ▸), liquids (Weinhardt *et al.*, 2010 ▸; Jeyachandran *et al.*, 2014 ▸; Yin *et al.*, 2015 ▸; Kunnus *et al.*, 2016 ▸) and gases (Weinhardt *et al.*, 2012 ▸; Fouda *et al.*, 2020 ▸; Kjellsson *et al.*, 2021 ▸), but have also been used to study applied material systems like solar cells (Hauschild *et al.*, 2021 ▸; Wilks *et al.*, 2021 ▸) or batteries (Wu *et al.*, 2020 ▸; Li *et al.*, 2023 ▸).

**Figure 3:**
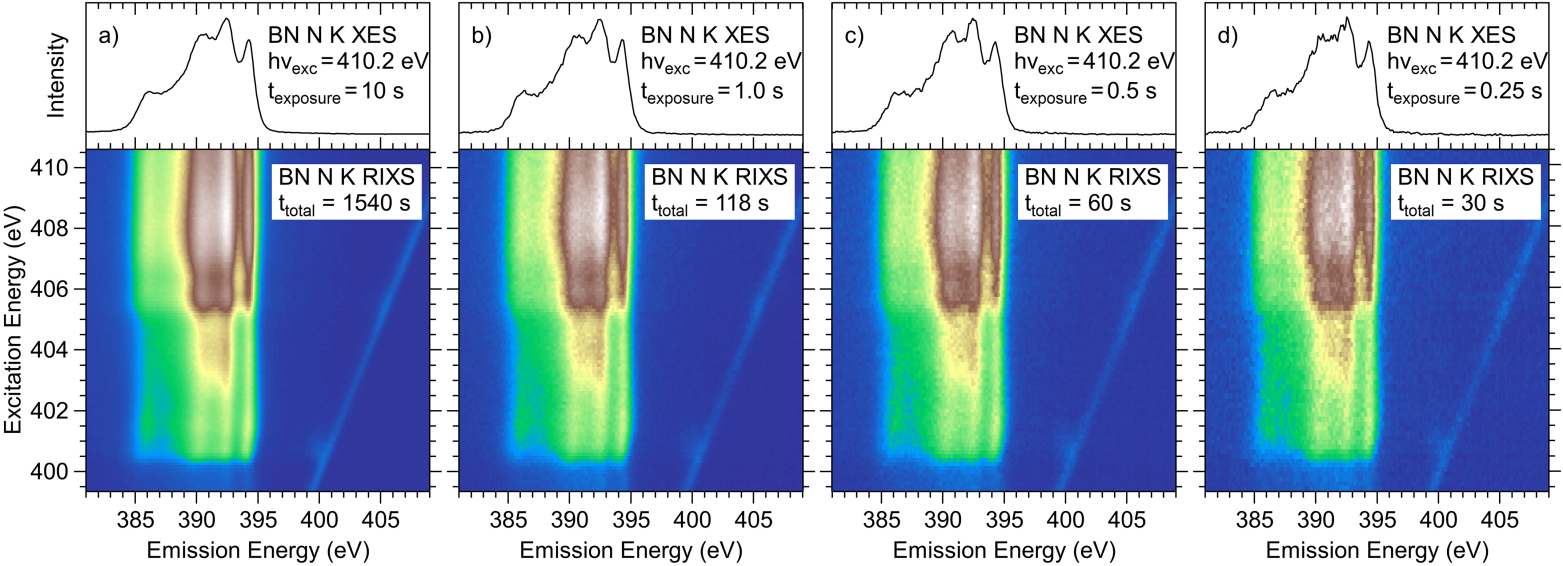
N *K* RIXS maps and non-resonant (*h*ν = 410.2 eV) XES spectra of *h*-BN measured in second diffraction order (*a*) with a step scan and (*b*)–(*d*) in continuous energy scans. The exposure times and total measurement times are given next to the spectra and RIXS maps, respectively.

For applied material systems, the required measurement times for these maps of a few ten minutes up to a few hours still limits the practical use of this approach. This limitation is now overcome by the new spectrometer design (in combination with the X-SPEC beamline) presented in this paper. Fig. 3 ▸(*a*) shows the RIXS map of a pressed *h*-BN pellet, measured with our spectrometer in a conventional step scan with an exposure time of ten seconds per spectrum, which leads to a total measurement time of slightly below half an hour. As can be seen, this map shows little to no noise, which suggests that (much) shorter measurement times are feasible. In a step scan, the practical gain of this, however, is limited by the time needed to move undulator and monochromator to the next energy, which is around 3.5 s in the present case. As discussed above, at X-SPEC we can use continuous scans to avoid this dead-time and, in combination with the short readout times of the CMOS sensor and the high spectrometer transmission, the rRIXS maps shown in Figs. 3 ▸(*b*)–3(*d*) become possible. These maps are collected in second diffraction order with total measurement times of 2, 1 and 0.5 min and exposure times of 1, 0.5 and 0.25 s per spectrum, respectively. At the top of each map, the corresponding XES spectrum, extracted from the map at an excitation energy of 410.2 eV, is shown. The X-SPEC beamline exit slit was set to 200 µm, giving an excitation energy width of ∼0.5 eV and a photon flux of ∼2 × 10^13^ photons s^−1^ at 100 mA ring current as estimated from the gold mesh current. Flux values and resolving powers of the X-SPEC beamline as a function of photon energy at smaller slit values are given by Weinhardt, Steininger *et al.* (2021 ▸). Table 2 ▸ lists the measurement parameters of the RIXS maps presented in Fig. 3 ▸. Switching from step scans to continuous scans strongly reduces the dead-time per point, and, even though the rRIXS maps in Figs. 3 ▸(*b*) and 3(*c*) are measured much faster, the dead-time *fraction* is lower than that of the step scan. Even for the fastest map, the dead-time still only accounts for 40% of the total measurement time.

**Figure 4:**
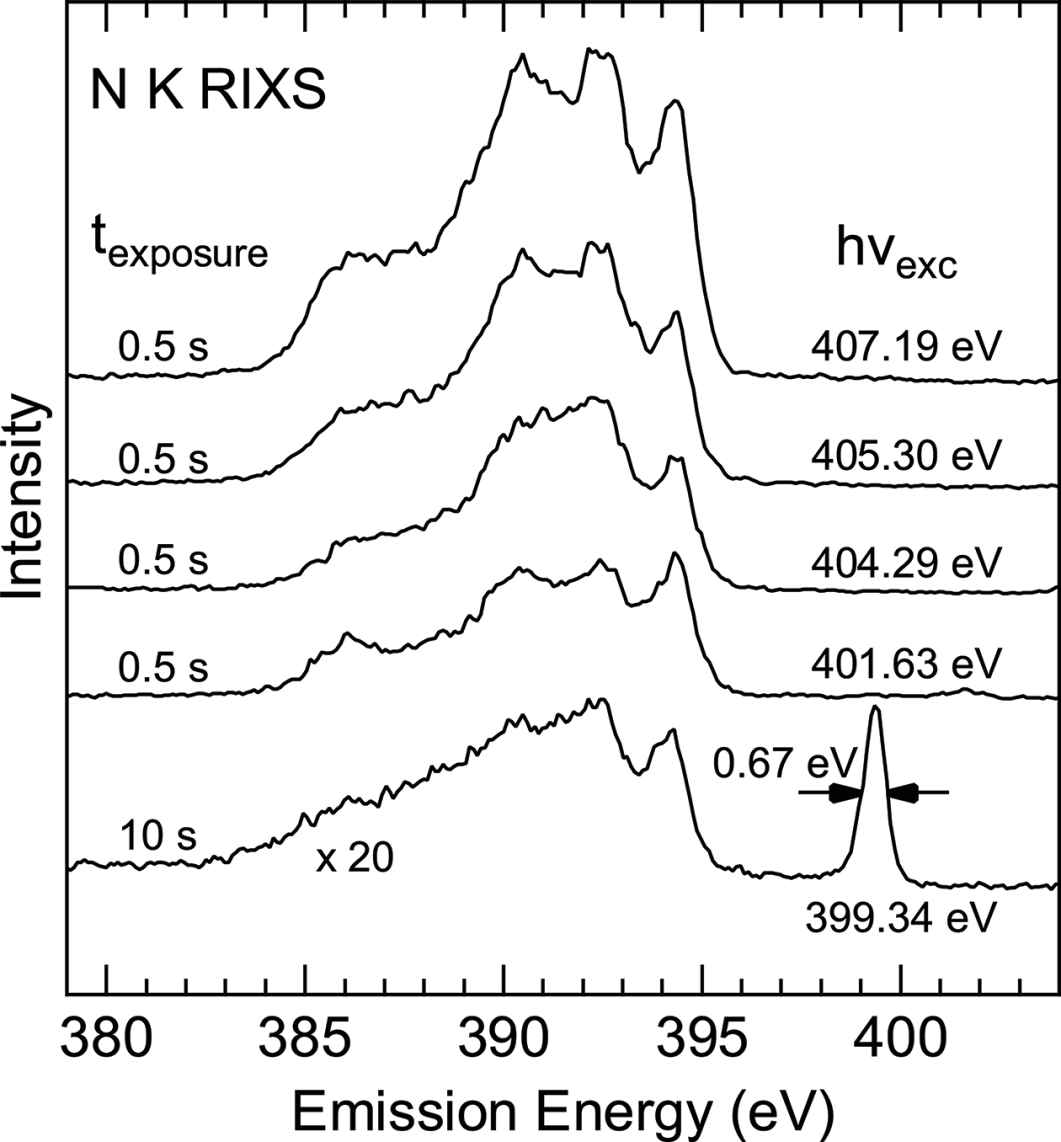
N *K* RIXS spectra extracted at selected energies from the maps in Figs. 3 ▸(*a*) (bottom-most spectrum, 399.34 eV) and 3(*c*). The bottom-most spectrum is excited below the N *K* absorption edge, only showing the elastically scattered line and emission excited with higher orders/harmonic of the beamline.

In Fig. 4 ▸, we show cuts from the RIXS maps at selected excitation energies. For comparison and benchmark, similar energies as in a recent experimental and theoretical RIXS study on *h*-BN (Vinson *et al.*, 2017 ▸) were chosen. Apart from some changes in relative peak intensities caused by the different scattering angle, our data agree well with that of Vinson *et al.* (2017 ▸). Remarkably, our data also exhibit a similar signal-to-noise ratio with exposure times of 0.5 s when compared with 1800–3600 s in Vinson *et al.* (2017 ▸). The bottom-most spectrum is excited below the absorption edge with some weak contributions from higher order/harmonics (∼5% of the first order/harmonic intensity) excitation and the elastically scattered photons. Evaluating the elastic peak, we find a combined full width at half-maximum (FWHM) of 0.67 eV, which includes both broadening from the beamline and the spectrometer. With a beamline contribution of ∼0.5 eV, the spectrometer resolution is estimated to 0.45 eV, limited by the spot size on the sample.

In addition to fast rRIXS maps, measurements at fixed energies can be performed with frequencies up to 5 Hz, limited by the dead-time of the control system. This can be useful for non-resonant excitation or at an excitation energy selected for best sensitivity to study a desired sample property in a time-resolved way. To further speed up the data collection, we are currently optimizing the control system reducing the acquisition dead-time of the *SPEC* scan significantly to be only limited by the maximal frequency of the CMOS camera (48 Hz), if the rRIXS signal strength is sufficiently high.

## 5. Summary

The design of a high-transmission spectrometer for rapid RIXS (rRIXS) maps is presented, together with a demonstration experiment of hexagonal boron nitride. With a compact design, maximized solid angle, optimized grating parameters, the use of a CMOS sensor tailored for soft X-rays, and a streamlined integration into the X-SPEC beamline control system, this spectrometer allows the total measurement time for a full rRIXS map to be reduced to half a minute in a continuous energy scan, with an exposure time of 0.25 s per spectrum. At the same time, the spectrometer achieves a resolving power *E*/Δ*E* of 1500 or better with its low-energy grating, and above 3000 with its high-energy grating. Furthermore, the spectrometer covers a very large energy range from 45 to 2000 eV with only two gratings by making use of higher-order reflections. With measurement times of well below 1 s per spectrum, the spectrometer opens the route to study chemical and electronic processes under operation conditions using rapid RIXS.

## Figures and Tables

**Table 1:** Optical parameters of the spectrometer

Element	Parameter	Low-energy grating (LEG)	High-energy grating (HEG)
Housing	Entrance arm length	400 mm	
	Exit arm length	1387–985 mm	1393–1002 mm

Grating	Dimensions	25 mm × 150 mm	
	Radius of curvature	19828 mm	25322 mm
	Slope error	<0.5 µrad	
	Grazing incidence angle	2.9–1.8°	1.0°
	Grazing exit angle	10.3–4.6°	4.8–2.8°
	Blaze angle	1.7°	0.9°
	Micro-roughness	<0.6 nm RMS	
	Central groove density	*a*_0_ = 600 mm^−1^	*a*_0_ = 1200 mm^−1^
	VLS parameters	*a*_1_ = 0.72306 mm^−2^	*a*_1_ = 0.63798 mm^−2^
		*a*_2_ = 7.9087 × 10^−6^ mm^−3^	*a*_2_ = 5.5736 × 10^−4^ mm^−3^
		*a*_3_ = 3.889 × 10^−6^ mm^−2^	*a*_3_ = 3.9074 × 10^−6^ mm^−2^
	Groove density	*n*(*x*) = *a*_0_ + *a*_1_*x* + *a*_2_*x*^2^ + *a*_3_*x*^3^	
	Maximum line density error	<0.1 mm^−1^	

Detector	Dimensions	22.5 mm × 22.5 mm	
	Pixel size	11.0 µm × 11.0 µm	

**Table 2:** Measurement parameters for the *h*-BN RIXS maps presented in Fig. 3 ▸

	Total time (s)	Exposure time per point (s)	Dead-time per point (s)	Dead-time fraction (%)	Step size (eV)	Number of spectra
Fig. 3 ▸(*a*)	1540	10	3.63	27	0.10	113
Fig. 3 ▸(*b*)	118	1	0.168	14	0.11	101
Fig. 3 ▸(*c*)	60	0.5	0.167	25	0.13	90
Fig. 3 ▸(*d*)	30	0.25	0.167	40	0.16	72

## References

[bb1] Ament, L. J. P., van Veenendaal, M., Devereaux, T. P., Hill, J. P. & van den Brink, J. (2011). *Rev. Mod. Phys.* **83**, 705–767.

[bb2] Blum, M., Weinhardt, L., Fuchs, O., Bär, M., Zhang, Y., Weigand, M., Krause, S., Pookpanratana, S., Hofmann, T., Yang, W., Denlinger, J. D., Umbach, E. & Heske, C. (2009). *Rev. Sci. Instrum.* **80**, 123102. 10.1063/1.3257926 20059126

[bb3] Callcott, T. A., Tsang, K. L., Zhang, C. H., Ederer, D. L. & Arakawa, E. T. (1986). *Rev. Sci. Instrum.* **57**, 2680–2690.

[bb4] Carlisle, J. A., Shirley, E. L., Hudson, E. A., Terminello, L. J., Callcott, T. A., Jia, J. J., Ederer, D. L., Perera, R. C. C. & Himpsel, F. J. (1995). *Phys. Rev. Lett.* **74**, 1234–1237. 10.1103/PhysRevLett.74.1234 10058968

[bb5] Chiuzbăian, S. G., Ghiringhelli, G., Dallera, C., Grioni, M., Amann, P., Wang, X., Braicovich, L. & Patthey, L. (2005). *Phys. Rev. Lett.* **95**, 197402. 10.1103/PhysRevLett.95.197402 16384022

[bb6] Chiuzbăian, S. G., Hague, C. F., Avila, A., Delaunay, R., Jaouen, N., Sacchi, M., Polack, F., Thomasset, M., Lagarde, B., Nicolaou, A., Brignolo, S., Baumier, C., Lüning, J. & Mariot, J.-M. (2014). *Rev. Sci. Instrum.* **85**, 043108. 10.1063/1.4871362 24784594

[bb7] Chuang, Y.-D., Feng, X., Glans-Suzuki, P.-A., Yang, W., Padmore, H. & Guo, J.-H. (2020). *J. Synchrotron Rad.* **27**, 695–707. 10.1107/S1600577520004440 PMC7206552 32381770

[bb8] Chuang, Y.-D., Pepper, J., McKinney, W., Hussain, Z., Gullikson, E., Batson, P., Qian, D. & Hasan, M. Z. (2005). *J. Phys. Chem. Solids*, **66**, 2173–2178.

[bb9] Cocco, D., Zangrando, M., Matteucci, M., Bondino, F., Platè, M., Zacchigna, M., Parmigiani, F., Nelles, B. & Prince, K. C. (2004). *AIP Conf. Proc.* **705**, 873–876.

[bb10] Dakovski, G. L., Lin, M.-F., Damiani, D. S., Schlotter, W. F., Turner, J. J., Nordlund, D. & Ogasawara, H. (2017). *J. Synchrotron Rad.* **24**, 1180–1186. 10.1107/S1600577517011869 29091061

[bb11] Dallera, C., Puppin, E., Trezzi, G., Incorvaia, N., Fasana, A., Braicovich, L., Brookes, N. B. & Goedkoop, J. B. (1996). *J. Synchrotron Rad.* **3**, 231–238. 10.1107/S0909049596006061 16702684

[bb12] Dvorak, J., Jarrige, I., Bisogni, V., Coburn, S. & Leonhardt, W. (2016). *Rev. Sci. Instrum.* **87**, 115109. 10.1063/1.4964847 27910402

[bb13] Eich, D., Fuchs, O., Groh, U., Weinhardt, L., Fink, R., Umbach, E., Heske, C., Fleszar, A., Hanke, W., Gross, E. K. U., Bostedt, C., Von Buuren, T., Franco, N., Terminello, L. J., Keim, M., Reuscher, G., Lugauer, H. & Waag, A. (2006). *Phys. Rev. B*, **73**, 115212.

[bb14] Ertan, E., Savchenko, V., Ignatova, N., Vaz da Cruz, V., Couto, R. C., Eckert, S., Fondell, M., Dantz, M., Kennedy, B., Schmitt, T., Pietzsch, A., Föhlisch, A., Gel’mukhanov, F., Odelius, M. & Kimberg, V. (2018). *Phys. Chem. Chem. Phys.* **20**, 14384–14397. 10.1039/c8cp01807c 29770402

[bb15] Fouda, A. E. A., Seitz, L. C., Hauschild, D., Blum, M., Yang, W., Heske, C., Weinhardt, L. & Besley, N. A. (2020). *J. Phys. Chem. Lett.* **11**, 7476–7482. 10.1021/acs.jpclett.0c01981 32787301

[bb16] Fuchs, O., Weinhardt, L., Blum, M., Weigand, M., Umbach, E., Bär, M., Heske, C., Denlinger, J., Chuang, Y. D., McKinney, W., Hussain, Z., Gullikson, E., Jones, M., Batson, P., Nelles, B. & Follath, R. (2009). *Rev. Sci. Instrum.* **80**, 063103. 10.1063/1.3133704 19566192

[bb17] Fuchs, O., Zharnikov, M., Weinhardt, L., Blum, M., Weigand, M., Zubavichus, Y., Bär, M., Maier, F., Denlinger, J. D., Heske, C., Grunze, M. & Umbach, E. (2008). *Phys. Rev. Lett.* **100**, 249802. 10.1103/PhysRevLett.100.027801 18232928

[bb18] Ghiringhelli, G., Matsubara, M., Dallera, C., Fracassi, F., Tagliaferri, A., Brookes, N. B., Kotani, A. & Braicovich, L. (2006). *Phys. Rev. B*, **73**, 035111.

[bb19] Ghiringhelli, G., Piazzalunga, A., Dallera, C., Trezzi, G., Braicovich, L., Schmitt, T., Strocov, V. N., Betemps, R., Patthey, L., Wang, X. & Grioni, M. (2006). *Rev. Sci. Instrum.* **77**, 113108.

[bb20] Götz, A., Taurel, E., Pons, J. E., Verdier, P., Chaize, J. M., Meyer, J., Poncet, F., Heunen, G. & Götz, E. (2003). *Proceedings of the 2003 International Conference on Accelerator and Large Experimental Physics Control Systems (ICALEPCS2003)*, 13–17 October 2003, Gyeongju, Korea, pp. 220–222. MP705.

[bb21] Harada, T., Teranishi, N., Watanabe, T., Zhou, Q., Bogaerts, J. & Wang, X. (2019). *Appl. Phys. Expr.* **13**, 016502.

[bb22] Harada, T., Teranishi, N., Watanabe, T., Zhou, Q., Yang, X., Bogaerts, J. & Wang, X. (2019). *Appl. Phys. Expr.* **12**, 082012.

[bb23] Hatsui, T., Setoyama, H., Shigemasa, E. & Kosugi, N. (2005). *J. Electron Spectrosc. Relat. Phenom.* **144–147**, 1059–1062.

[bb24] Hauschild, D., Seitz, L., Gharibzadeh, S., Steininger, R., Jiang, N., Yang, W., Paetzold, U. W., Heske, C. & Weinhardt, L. (2021). *Appl. Mater. Interfaces*, **13**, 53202–53210. 10.1021/acsami.1c15707 34709800

[bb25] Henke, B. L., Gullikson, E. M. & Davis, J. C. (1993). *At. Data Nucl. Data Tables*, **54**, 181–342.

[bb26] Hennies, F., Pietzsch, A., Berglund, M., Föhlisch, A., Schmitt, Th., Strocov, V., Karlsson, H. O., Andersson, J. & Rubensson, J.-E. (2010). *Phys. Rev. Lett.* **104**, 193002. 10.1103/PhysRevLett.104.193002 20866962

[bb27] Heske, C., Groh, U., Fuchs, O., Weinhardt, L., Umbach, E., Schedel-Niedrig, T., Fischer, C. H., Lux-Steiner, M. C., Zweigart, S., Niesen, T. P., Karg, F., Denlinger, J. D., Rude, B., Andrus, C. & Powell, F. (2003). *J. Chem. Phys.* **119**, 10467–10470.

[bb28] Huang, H. Y., Chen, Z. Y., Wang, R.-P., de Groot, F. M. F., Wu, W. B., Okamoto, J., Chainani, A., Singh, A., Li, Z.-Y., Zhou, J.-S., Jeng, H.-T., Guo, G. Y., Park, J.-G., Tjeng, L. H., Chen, C. T. & Huang, D. J. (2017). *Nat. Commun.* **8**, 15929. 10.1038/ncomms15929 PMC5493765 28660878

[bb29] Jeyachandran, Y. L., Meyer, F., Nagarajan, S., Benkert, A., Bär, M., Blum, M., Yang, W., Reinert, F., Heske, C., Weinhardt, L. & Zharnikov, M. (2014). *J. Phys. Chem. Lett.* **5**, 4143–4148. 10.1021/jz502186a 26278946

[bb30] Johnson, P. D. & Ma, Y. (1994). *Phys. Rev. B*, **49**, 5024–5027. 10.1103/physrevb.49.5024 10011438

[bb31] Kjellsson, L., Ekholm, V., Agåker, M., Såthe, C., Pietzsch, A., Karlsson, H. O., Jaouen, N., Nicolaou, A., Guarise, M., Hague, C., Lüning, J., Chiuzbăian, S. G. & Rubensson, J.-E. (2021). *Phys. Rev. A*, **103**, 022812.

[bb32] Kunnus, K., Zhang, W., Delcey, M. G., Pinjari, R. V., Miedema, P. S., Schreck, S., Quevedo, W., Schröder, H., Föhlisch, A., Gaffney, K. J., Lundberg, M., Odelius, M. & Wernet, P. (2016). *J. Phys. Chem. B*, **120**, 7182–7194. 10.1021/acs.jpcb.6b04751 27380541

[bb33] Lee, S.-J., Titus, C. J., Alonso Mori, R., Baker, M. L., Bennett, D. A., Cho, H.-M., Doriese, W. B., Fowler, J. W., Gaffney, K. J., Gallo, A., Gard, J. D., Hilton, G. C., Jang, H., Joe, Y. I., Kenney, C. J., Knight, J., Kroll, T., Lee, J.-S., Li, D., Lu, D., Marks, R., Minitti, M. P., Morgan, K. M., Ogasawara, H., O’Neil, G. C., Reintsema, C. D., Schmidt, D. R., Sokaras, D., Ullom, J. N., Weng, T.-C., Williams, C., Young, B. A., Swetz, D. S., Irwin, K. D. & Nordlund, D. (2019). *Rev. Sci. Instrum.* **90**, 113101. 10.1063/1.5119155 31779391

[bb34] Léon, A., Fiedler, A., Blum, M., Benkert, A., Meyer, F., Yang, W., Bär, M., Scheiba, F., Ehrenberg, H., Weinhardt, L. & Heske, C. (2017). *J. Phys. Chem. C*, **121**, 5460–5466.

[bb35] Li, B., Zhuo, Z., Zhang, L., Iadecola, A., Gao, X., Guo, J., Yang, W., Morozov, A. V., Abakumov, A. M. & Tarascon, J.-M. (2023). *Nat. Mater.* **22**, 1370–1379. 10.1038/s41563-023-01679-x 37798516

[bb36] Lu, H., Rossi, M., Nag, A., Osada, M., Li, D. F., Lee, K., Wang, B. Y., Garcia-Fernandez, M., Agrestini, S., Shen, Z. X., Been, E. M., Moritz, B., Devereaux, T. P., Zaanen, J., Hwang, H. Y., Zhou, K.-J. & Lee, W. S. (2021). *Science*, **373**, 213–216. 10.1126/science.abd7726 34244413

[bb37] Marschall, F., Yin, Z., Rehanek, J., Beye, M., Döring, F., Kubiček, K., Raiser, D., Veedu, S. T., Buck, J., Rothkirch, A., Rösner, B., Guzenko, V. A., Viefhaus, J., David, C. & Techert, S. (2017). *Sci. Rep.* **7**, 8849. 10.1038/s41598-017-09052-0 PMC5562906 28821805

[bb38] Meisel, A., Steuer, I. & Szargan, R. (1968). *At. Spectrosc.* **23**, 527–533.

[bb39] Meyer, F., Blum, M., Benkert, A., Hauschild, D., Nagarajan, S., Wilks, R. G., Andersson, J., Yang, W., Zharnikov, M., Bär, M., Heske, C., Reinert, F. & Weinhardt, L. (2014). *J. Phys. Chem. B*, **118**, 13142–13150. 10.1021/jp5089417 25341188

[bb40] Nagasaka, M., Hatsui, T., Horigome, T., Hamamura, Y. & Kosugi, N. (2010). *J. Electron Spectrosc. Relat. Phenom.* **177**, 130–134.

[bb41] Nordgren, J. & Nyholm, R. (1986). *Nucl. Instrum. Methods Phys. Res. A*, **246**, 242–245.

[bb42] O’Bryan, H. M. & Skinner, H. W. B. (1940). *Proc. R. Soc. London*, **176**, 229–262.

[bb43] Qiao, R., Li, Q., Zhuo, Z., Sallis, S., Fuchs, O., Blum, M., Weinhardt, L., Heske, C., Pepper, J., Jones, M., Brown, A., Spucces, A., Chow, K., Smith, B., Glans, P.-A., Chen, Y., Yan, S., Pan, F., Piper, L. F. J., Denlinger, J., Guo, J., Hussain, Z., Chuang, Y.-D. & Yang, W. (2017). *Rev. Sci. Instrum.* **88**, 033106. 10.1063/1.4977592 28372380

[bb44] Roychoudhury, S., Zhuo, Z., Qiao, R., Wan, L., Liang, Y., Pan, F., Chuang, Y., Prendergast, D. & Yang, W. (2021). *Appl. Mater. Interfaces*, **13**, 45488–45495. 10.1021/acsami.1c11970 34529403

[bb45] Rubensson, J.-E., Söderström, J., Binggeli, C., Gråsjö, J., Andersson, J., Såthe, C., Hennies, F., Bisogni, V., Huang, Y., Olalde, P., Schmitt, T., Strocov, V. N., Föhlisch, A., Kennedy, B. & Pietzsch, A. (2015). *Phys. Rev. Lett.* **114**, 133001. 10.1103/PhysRevLett.114.133001 25884123

[bb46] Schäfers, F. (2008). *Modern Developments in X-ray and Neutron Optics*, Vol. 137 of *Springer Series in Optical Sciences*, p. 9. Berlin/Heidelberg: Springer.

[bb47] Schlappa, J., Wohlfeld, K., Zhou, K. J., Mourigal, M., Haverkort, M. W., Strocov, V. N., Hozoi, L., Monney, C., Nishimoto, S., Singh, S., Revcolevschi, A., Caux, J.-S., Patthey, L., Rønnow, H. M., van den Brink, J. & Schmitt, T. (2012). *Nature*, **485**, 82–85. 10.1038/nature10974 22522933

[bb48] Schunck, J. O., Döring, F., Rösner, B., Buck, J., Engel, R. Y., Miedema, P. S., Mahatha, S. K., Hoesch, M., Petraru, A., Kohlstedt, H., Schüssler-Langeheine, C., Rossnagel, K., David, C. & Beye, M. (2021). *Optica*, **8**, 156–160.

[bb49] Schwanke, C., Golnak, R., Xiao, J. & Lange, K. M. (2014). *Rev. Sci. Instrum.* **85**, 103120. 10.1063/1.4899063 25362384

[bb50] Seitz, L. C., Doronkin, D. E., Hauschild, D., Casapu, M., Zengel, D., Zimina, A., Kreikemeyer-Lorenzo, D., Blum, M., Yang, W., Grunwaldt, J.-D., Heske, C. & Weinhardt, L. (2022). *J. Phys. Chem. C*, **126**, 20998–21009.

[bb51] Shin, S., Agui, A., Fujisawa, M., Tezuka, Y., Ishii, T. & Hirai, N. (1995). *Rev. Sci. Instrum.* **66**, 1584–1586.

[bb52] Strocov, V. N., Schmitt, T., Flechsig, U., Patthey, L. & Chiuzbăian, G. S. (2011). *J. Synchrotron Rad.* **18**, 134–142. 10.1107/S0909049510054452 PMC3133478 21335898

[bb53] Tokushima, T., Harada, Y., Ohashi, H., Senba, Y. & Shin, S. (2006). *Rev. Sci. Instrum.* **77**, 063107. 10.1063/1.3680559 22299938

[bb54] Tokushima, T., Horikawa, Y. & Shin, S. (2011). *Rev. Sci. Instrum.* **82**, 073108. 10.1063/1.3610454 21806173

[bb55] Vale, J. G., Dashwood, C. D., Paris, E., Veiga, L. S. I., Garcia-Fernandez, M., Nag, A., Walters, A., Zhou, K.-J., Pietsch, I.-M., Jesche, A., Gegenwart, P., Coldea, R., Schmitt, T. & McMorrow, D. F. (2019). *Phys. Rev. B*, **100**, 224303.

[bb56] Vinson, J., Jach, T., Müller, M., Unterumsberger, R. & Beckhoff, B. (2017). *Phys. Rev. B*, **96**, 205116. 10.1103/PhysRevB.96.205116 PMC5766010 29333524

[bb57] Warwick, T., Chuang, Y.-D., Voronov, D. L. & Padmore, H. A. (2014). *J. Synchrotron Rad.* **21**, 736–743. 10.1107/S1600577514009692 24971968

[bb58] Weinhardt, L., Benkert, A., Meyer, F., Blum, M., Hauschild, D. G., Wilks, R., Bär, M., Yang, W., Zharnikov, M., Reinert, F. & Heske, C. (2019). *Phys. Chem. Chem. Phys.* **21**, 13207–13214. 10.1039/c9cp02481f 31179459

[bb59] Weinhardt, L., Benkert, A., Meyer, F., Blum, M., Wilks, R. G., Yang, W., Bär, M., Reinert, F. & Heske, C. (2012). *J. Chem. Phys.* **136**, 144311. 10.1063/1.3702644 22502522

[bb60] Weinhardt, L., Blum, M., Bär, M., Heske, C., Cole, B., Marsen, B. & Miller, E. L. (2008). *J. Phys. Chem. C*, **112**, 3078–3082.

[bb61] Weinhardt, L., Fuchs, O., Blum, M., Bär, M., Weigand, M., Denlinger, J. D., Zubavichus, Y., Zharnikov, M., Grunze, M., Heske, C. & Umbach, E. (2010). *J. Electron Spectrosc. Relat. Phenom.* **177**, 206–211.

[bb62] Weinhardt, L., Fuchs, O., Fleszar, A., Bär, M., Blum, M., Weigand, M., Denlinger, J. D., Yang, W., Hanke, W., Umbach, E. & Heske, C. (2009). *Phys. Rev. B*, **79**, 165305.

[bb63] Weinhardt, L., Hauschild, D., Steininger, R., Jiang, N., Blum, M., Yang, W. & Heske, C. (2021). *Anal. Chem.* **93**, 8300–8308. 10.1021/acs.analchem.1c01187 34076421

[bb64] Weinhardt, L., Steininger, R., Kreikemeyer-Lorenzo, D., Mangold, S., Hauschild, D., Batchelor, D., Spangenberg, T. & Heske, C. (2021). *J. Synchrotron Rad.* **28**, 609–617. 10.1107/S1600577520016318 PMC7941287 33650573

[bb65] Werme, L., Grennberg, B., Nordgren, J., Nordling, C. & Siegbahn, K. (1973). *Nature*, **242**, 453–455.

[bb66] Wilks, R. G., Erbing, A., Sadoughi, G., Starr, D. E., Handick, E., Meyer, F., Benkert, A., Iannuzzi, M., Hauschild, D., Yang, W., Blum, M., Weinhardt, L., Heske, C., Snaith, H. J., Odelius, M. & Bär, M. (2021). *J. Phys. Chem. Lett.* **12**, 3885–3890. 10.1021/acs.jpclett.1c00745 33856793

[bb67] Wilks, R. G., MacNaughton, J. B., Kraatz, H.-B., Regier, T., Blyth, R. I. R. & Moewes, A. (2009). *J. Phys. Chem. A*, **113**, 5360–5366. 10.1021/jp900794v 19402716

[bb68] Wu, J., Yang, Y. & Yang, W. (2020). *Dalton Trans.* **49**, 13519–13527. 10.1039/d0dt01782e 32785340

[bb69] Yamane, H., Kosugi, N. & Hatsui, T. (2013). *J. Electron Spectrosc. Relat. Phenom.* **188**, 155–160.

[bb70] Yin, Z., Rajkovic, I., Thekku Veedu, S., Deinert, S., Raiser, D., Jain, R., Fukuzawa, H., Wada, S., Quevedo, W., Kennedy, B., Schreck, S., Pietzsch, A., Wernet, P., Ueda, K., Föhlisch, A. & Techert, S. (2015). *Z. Phys. Chem.* **229**, 1855–1867.

[bb71] Zhou, K.-J., Walters, A., Garcia-Fernandez, M., Rice, T., Hand, M., Nag, A., Li, J., Agrestini, S., Garland, P., Wang, H., Alcock, S., Nistea, I., Nutter, B., Rubies, N., Knap, G., Gaughran, M., Yuan, F., Chang, P., Emmins, J. & Howell, G. (2022). *J. Synchrotron Rad.* **29**, 563–580. 10.1107/S1600577522000601 PMC8900866 35254322

[bb72] Zubavichus, Y., Fuchs, O., Weinhardt, L., Heske, C., Umbach, E., Denlinger, J. D. & Grunze, M. (2004). *Radiat. Res.* **161**, 346–358. 10.1667/rr3114.1 15108703

